# Supplementary material for: A Global Screen for Assembly State Changes of the Mitotic Proteome by SEC-SWATH-MS
Source: Cell Syst. 2020 Feb 26;10(2):133–155.e6. doi: 10.1016/j.cels.2020.01.001 (PMC7042714; doi:10.1016/j.cels.2020.01.001)

Q0PNE2 | ELP6_HUMAN | ELP6 ATP1 C3orf75 TMEM103
Monomer MW [kDa]: 29.793 Monomer expected elution fraction: 51

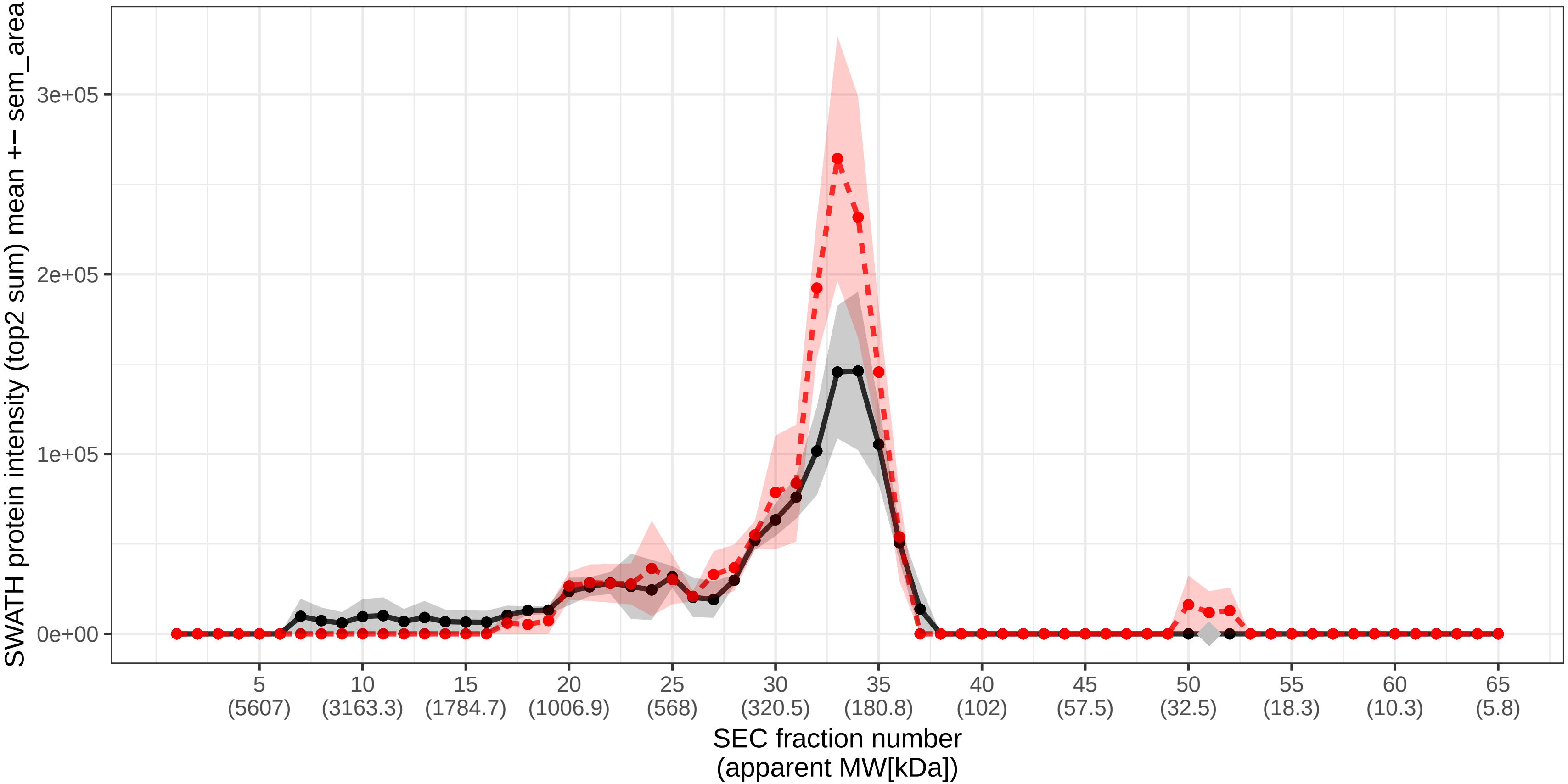

Supplement: Data S1. SEC-SWATH-MS Protein Chromatograms, Related to Figure 1 [file mmc6.zip › SECchrom_Q0PNE2_ELP6_HUMAN_ELP6_ATP1_C3orf75_TMEM103.pdf]
